# The complex interplay between risk tolerance and the spread of infectious diseases

**DOI:** 10.1098/rsif.2024.0486

**Published:** 2025-04-23

**Authors:** Maximilian M. Nguyen, Ari S. Freedman, Matthew A. Cheung, Chadi M. Saad-Roy, Baltazar Espinoza, Bryan T. Grenfell, Simon A. Levin

**Affiliations:** ^1^ Lewis-Sigler Institute, Princeton University, Princeton, NJ, USA; ^2^ Department of Ecology and Evolutionary Biology, Princeton University, Princeton, NJ, USA; ^3^ Program in Applied and Computational Mathematics, Princeton University, Princeton, NJ, USA; ^4^ Miller Institute for Basic Research in Science, University of California Berkeley, Berkeley, CA, USA; ^5^ Department of Integrative Biology, University of California, Berkeley, CA, USA; ^6^ Biocomplexity Institute, University of Virginia, Charlottesville, VA, USA

**Keywords:** risk tolerance, epidemic size, intervention adoption and usage, heterogeneous behaviour

## Abstract

Risk-driven behaviour provides a feedback mechanism through which individuals both shape and are collectively affected by an epidemic. We introduce a general and flexible compartmental model to study the effect of heterogeneity in the population with regard to risk tolerance. The interplay between behaviour and epidemiology leads to a rich set of possible epidemic dynamics. Depending on the behavioural composition of the population, we find that increasing heterogeneity in risk tolerance can either increase or decrease the epidemic size. We find that multiple waves of infection can arise due to the interplay between transmission and behaviour, even without the replenishment of susceptibles. We find that increasing protective mechanisms such as the effectiveness of interventions, the fraction of risk-averse people in the population and the duration of intervention usage reduce the epidemic overshoot. When the protection is pushed past a critical threshold, the epidemic dynamics enter an underdamped regime where the epidemic size exactly equals the herd immunity threshold and overshoot is eliminated. Finally, we can find regimes where epidemic size does not monotonically decrease with a population that becomes increasingly risk-averse.

## 1. Introduction

Recent outbreaks such as the COVID-19 pandemic, the 2014 Ebola outbreak, the 2009 influenza A (H1N1) pandemic and the 2002 SARS epidemic brought to light many of the challenges of mounting an effective and unified epidemic response in a country as large and as diverse as the United States. Particularly during the COVID-19 pandemic, people were split in opinion on questions such as the origin of the virus [1], whether they would social distance or wear a mask [2–4] or whether the country should even have a pandemic response at all [5]. As time progressed, the situation became more dire and the death toll accumulated. People then had a new battery of questions to address, such as whether or not they would adhere to mandatory lockdowns [6–8] or whether they felt comfortable using the new mRNA vaccines [9,10]. Compounding the issue were the multiple streams of information and potential misinformation spread through social media and other channels [11–14]. People’s stances on the questions and issues were diverse, arising from the milieu of differences in culture, geography, scientific education, sources of information, political leanings and individual identity [15–17].

Taken altogether, these differences within the population reflect a spectrum of people’s risk tolerances to a circulating infectious disease. For any given intervention, such as social distancing, wearing a mask or taking a vaccine, each person in the population falls somewhere on a spectrum of willingness to adopt the intervention. Given a threat level of an infectious disease in the population, some people will readily wear masks, whereas other people will refuse to.

In this study, we aim to analyse the impact of heterogeneity in risk tolerance and the resulting behavioural response on the dynamics of epidemics. We seek to add to a burgeoning literature on the impact of human behaviour in epidemic response [18], which the recent pandemic highlighted as an area for further exploration in preparation for the next large-scale global health crisis [19–21]. To study the impact of heterogeneity in risk tolerance on epidemic dynamics, we introduce a simple and flexible modelling framework based on ordinary differential equations that can be used for different interventions and an arbitrary partitioning of the population with regard to risk tolerance and behavioural responses. We will examine and discuss potential interesting outcomes that can arise from coupling individual-level preferences and population-level epidemiology.

Under the existing classification scheme for behaviour–disease models [22,23], to formulate a model we have to make assumptions about the source of information that leads to behaviour changes, the type of information and the effect of the behaviour change on the dynamics of disease spread. First, to remove any spatial or local effects that might obscure the effect of risk tolerance, we choose the simplest assumption for the source of information and assume that information is globally available to all individuals in the population. Second, we had to make an assumption about the type of information that individuals are considering. In reality, people’s behaviours arise due to a complex amalgam of factors and considerations. Here, we have greatly simplified the behavioural response by ascribing individuals with a characteristic called risk tolerance, which encapsulates fear and other underlying biopsychosocial factors. We assume that risk tolerance is driven solely by information pertaining to the epidemic dynamics, in particular the case prevalence. In the electronic supplementary material, we also consider a model that relies on incidence information as the basis for behaviour changes instead. Finally, we had to make an assumption on the effect of behaviour change on the spread of disease. While the model classification scheme of Funk *et al*. [22] makes the distinction between a behaviour change that results in a change in the individual’s state (e.g. susceptible to immune via vaccination) and a change that results in a change in transmission parameters (such as a decrease in transmission rate), the modelling framework introduced here allows for both scenarios to be considered based on the choice of intervention effectiveness.

While the literature contains a number of models that feature global information of prevalence, our model here diverges on several fronts. Here, we have chosen not to consider the complexities that might have been taken by incorporating more economic or game-theoretic considerations [24–31]. The main novel feature of our model is the consideration of heterogeneity in the population, allowing for the population to be split into arbitrary factions of risk tolerance [32–36]. The model here also allows for people to remove protection at a rate independent of the disease [23]. A key advantage of the model framework presented here lies in its inherent simplicity, having only four main types of compartments and no time-dependent parameters, making the model more amenable to analysis.

## 2. Results

### 2.1. Model of adaptive intervention usage under heterogeneous risk tolerance

Here, we assume people’s risk-aversion manifests as the rate at which they adopt individual interventions in response to an infectious disease outbreak. The intuition underlying this paradigm is that more risk-averse individuals are more sensitive to becoming sick and thus will adopt interventions at a faster rate than more risk-tolerant people. We consider the following SPIR compartmental model of a population with n differing levels of risk tolerance (2.1)–(2.4). This model features four types of classes: unprotected susceptible (S), protected susceptible (P), infectious (I) and recovered with permanent immunity (R). Since there are n differing levels of risk tolerance, we subdivide the susceptible population into n discrete groups indexed by i, where i∈{1,2,...,n}. Each tolerance level is characterized by an intervention adoption rate parameter ($\lambda_{i}$) and an intervention relaxation rate parameter ($\delta_{i}$). Transitions of susceptibles between their unprotected class ($S_{i}$) and their corresponding protected class ($P_{i}$) are governed by the corresponding parameters of the same index ($\lambda_{i},\delta_{i}$). Overall, the system is governed by 3+2n parameters: a transmission rate parameter (β), a recovery rate parameter (γ), an intervention effectiveness parameter (ϵ) and an intervention adoption rate ($\lambda_{i}$) and intervention relaxation rate ($\delta_{i}$) for each tolerance level:


(2.1)dSidt=−βSiI−λiSiI+δiPi,(2.2)dPidt=−(1−ϵ)βPiI−δiPi+λiSiI,(2.3)dIdt=−γI+∑i=1n(βSiI+(1−ϵ)βPiI),(2.4)dRdt=γI.

The transition from the unprotected susceptible state to the protected susceptible state represents individuals implementing an intervention that confers them protection against disease transmission from an infected individual. The rate at which intervention adoption occurs may be driven by individuals considering information such as the epidemic incidence rate (e.g. cases per day), the total number of infected individuals in the population (e.g. total number of active cases) and mortality rate (e.g. deaths per day) [34]. Here, we assume that individuals have knowledge about the total fraction of infected individuals (I) and respond accordingly. Parameterizing each person’s individual risk tolerance by $\lambda_{i}$, we assume each individual person adopts an intervention at a rate $\lambda_{i}I$. Then, if there are $S_{i}$ fraction of people that behave exactly the same (i.e. have the same level of risk-aversion), then at the population scale there is a collective adoption rate of $\lambda_{i}S_{i}I$. The same reasoning holds for each of the n tolerance levels. We also consider a model where the adoption rate is driven by individuals reacting to the incidence rate (electronic supplementary materials); while this produces a more complex mathematical model, the results are qualitatively similar.

The effectiveness of the intervention being used is captured by the parameter ϵ, which linearly scales down the transmission rate between infected and protected susceptibles. In the limit of ϵ=1, the intervention is perfectly effective and protected individuals cannot become infected. In the limit of ϵ=0 then the intervention is completely ineffective, which reduces the model to an SIR model without interventions. For simplicity, we assume each epidemic features only a single type of intervention (whether that be masking, social distancing, vaccines, etc.) and that the effectiveness of an intervention is identical across the population. In reality, multiple interventions may be available concurrently, which would drive additional variation in behaviour due to differences in risk sensitivity across the population.

This model allows for protected individuals to relax their usage of interventions, becoming unprotected in the process. Here, individuals in the protected class can relax back to the unprotected class through an infection-independent rate ($\delta_{i}P_{i}$) that is governed by the intervention relaxation rate parameter ($\delta_{i}$) for each tolerance level. In the limit of $\delta_{i}=0$, an intervention is irreversible, which would represent an intervention such as vaccines with permanent immunity. When $\delta_{i}$ is non-zero, individuals are using interventions such as masking or social distancing. This relaxation rate is motivated by factors such as psychological fatigue of social distancing [37,38] and physical discomfort with wearing masks [39]. In general, we will consider the regime where the relaxation rate δ is of comparable scale or smaller than the transmission scale (i.e. δ≤β). This reflects the intuition that people are likely to continue to protect themselves with interventions even beyond an initial outbreak [40]. Since we always assume there are no protected individuals initially, the dynamics of the infected compartment is initially only a function of the unprotected transmission and recovery parameters.

An important parameter that governs the outbreak dynamics is the basic reproduction number ($R_{0}$), which can be derived using the next-generation matrix method [41]. For this model, if one assumes there are no initially protected individuals (i.e. $P_{i}=0$) and that I is initially small, then $R_{0}=\frac{\beta }{\gamma }$.

**Figure 1.**
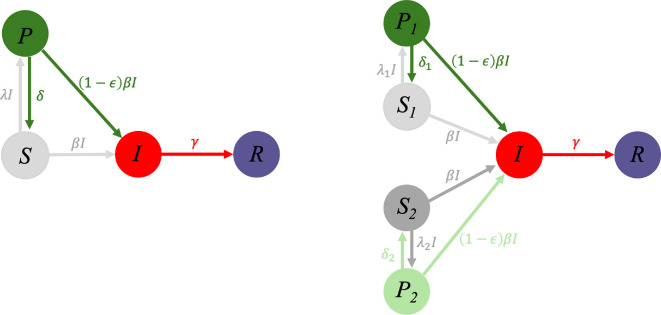
Flow diagram for an SIR model with adaptive interventions for either (left) a population with homogeneous risk tolerance or (right) a heterogeneous population with two different levels of risk tolerance. Susceptible individuals can access a more protected susceptible state through usage of interventions. The transition rate to the protected state depends on the incidence level. The protected state offers a 1−ϵ reduction in transmission rate over the normal susceptible state.

For simplicity, we consider the model for the case when n=1 and n=2. A schematic for these two cases is shown in figure 1. However, the framework is general and can be extended to any discrete number of groups.

For convention, when there are two susceptible classes, we assume the first susceptible class ($S_{1}$) has a lower risk tolerance for becoming infected (i.e. more risk-averse). As a result, these individuals more readily adopt the intervention (i.e. $\lambda_{1}>\lambda_{2}$), making individuals in this class transition more rapidly to the protected susceptible state ($P_{1}$). The second susceptible class ($S_{2}$) is more risk tolerant (i.e. more risk-taking), and thus is less eager to use the intervention, making individuals in this class transition more slowly to their protected susceptible state ($P_{2}$).

#### 2.1.1. Adaptive adoption of interventions can produce damped oscillations

**Figure 2.**
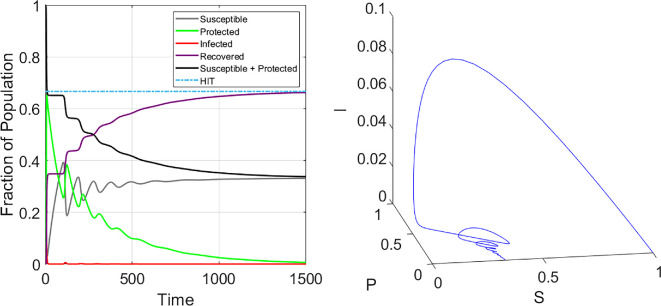
Left: Time series for population with homogeneous risk tolerance and adaptive intervention usage indicate the possibility for damped oscillations. Parameter values: β=3,γ=1,ϵ=0.8,λ=10,δ=0.01. Initial conditions: $I(0)=10^{-6},S(0)=1-I(0),P(0)=R(0)=0$. The HIT is given by $1-\frac{\gamma }{\beta }$. Right: The corresponding phase space trajectory for the dynamics shown in the left panel.

The coupling of intervention usage to the incidence rate and the resulting adaptive changes enables the epidemic dynamics to display a much richer set of behaviour over the simple SIR model. From figure 2, we see this particular set of conditions can deterministically produce multiple waves of infection, even when vital dynamics (i.e. birth and death processes) are not considered.

Evidence for cycling of individuals between using interventions and not using interventions during the COVID pandemic can be seen in longitudinal usage data [42–45]. The possibility for these oscillations highlights the intimate connection between individual human behaviour and intervention usage in shaping the dynamics of epidemics, while also be affected by the collective decision of everyone in the population. The coupling of behaviour and epidemiology here provides a feedback mechanism where an increasing incidence rate prompts more individuals to adopt an intervention, which lowers the overall incidence rate; however, as the epidemic wanes and factors such as fatigue or discomfort set in, people begin dropping their usage of interventions, which may eventually lead to another wave of outbreaks if enough people become unprotected while infected individuals still remain, and then the cycle can be repeated. The oscillatory phenomenology here is reminiscent of several other behavioural models in the literature [46,47].

#### 2.1.2. Protective mechanisms saturate in underdamped regime that eliminates epidemic overshoot

One might have the intuition that having more people that will more readily adopt an intervention (i.e. mask, social distance or vaccinate) or increasing the effectiveness of the intervention in reducing transmission will further decrease the size of the epidemic. Here, we define epidemic size to be the cumulative fraction of infections over the course of the epidemic, or equivalently, the total depletion of susceptible people over the course of the epidemic. While we find this intuition to be mostly correct, we unexpectedly find that the protection conferred by either of these mechanisms can saturate once a critical parameter threshold has been passed.

**Figure 3.**
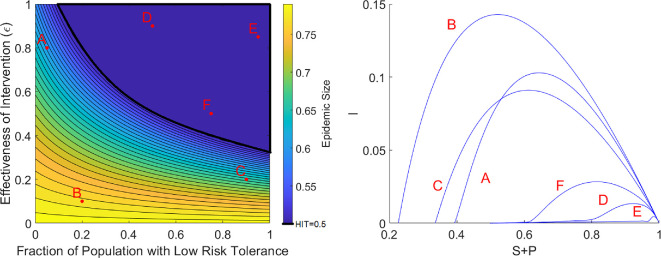
Left: Epidemic size as a function of varying the fraction of the population that are low-risk tolerance (i.e. those with higher λ). The colour bar indicates the epidemic size. The region outlined in black indicates the area described by the underdamped regime. The HIT is given by $1-\frac{\gamma }{\beta }.$ Right: Corresponding orbits in the *I* versus *S* + *P* plane for the sampled points in parameter space. Parameter values: $\beta =2,\gamma =1,\lambda_{1}=100,\delta_{1}=0.1,\lambda_{2}=1,\delta_{2}=0.1$. Initial conditions: $I(0)=10^{-8},P_{1}(0)=P_{2}(0)=R(0)=0$.

In figure 3, left, we see that increasing the effectiveness of the intervention or increasing the fraction of the population that are risk-averse monotonically decreases the epidemic size. However, in the dark blue region (which we will refer to as the underdamped regime) where both protection mechanisms are at their highest, we see no further reduction in the epidemic size. In this region, the final epidemic size is 0.5. Coincidentally, since $R_{0}=2$ for this figure, the herd immunity threshold (HIT), which is the fraction of susceptibles that must be eliminated from the pool to prevent an outbreak from growing and is given by the formula $1-\frac{1}{R_{0}}$, is also 0.5. This equality between epidemic size and HIT does not happen for the Kermack–McKendrick SIR model, which is a model with no behavioural component. Since the epidemic size is the sum of the herd immunity threshold and the epidemic overshoot, this implies that epidemic overshoot is eliminated in the underdamped regime. Intuition regarding the underdamped regime can be seen from a proof that the epidemic size equals HIT (implying epidemic overshoot is eliminated) for a one-group model in the underdamped regime (electronic supplementary material).

The orbits of the dynamics from different areas of this parameter space are shown in figure 3, right. We note that orbits in the overdamped region (Orbits A, B, C) are parabolas and that each of those orbits ends at a different final fraction of susceptibles. In contrast, orbits in the underdamped regime (Orbits D, E, F) are qualitatively different in that they display long tails that converge to the same final fraction of susceptibles. We see that even though the final epidemic size is the same throughout the underdamped region, the trajectories to reach the same final epidemic size can look qualitatively different.

Electronic supplementary material, figures S4 and S5, show a larger sampling of trajectories if one fixes either the fraction of the population with low risk tolerance or the intervention effectiveness respectively. It becomes clear that at the border of the underdamped region, we can see a clear change in the qualitative behaviour of the trajectories as the threshold is crossed.

However, we should make the point that this is not evidence that highly effective interventions are a waste or that the overall population should tolerate risky behaviour. As this is a model with a large parameter space, we cannot visualize all of it. If we could, we would find many parameter regimes where the protection mechanisms never reach a critical threshold, which implies the conventional intuition of increasing intervention effectiveness and having more risk-averse people being beneficial applies.

#### 2.1.3. The threshold to the underdamped regime is reduced when intervention usage is prolonged

**Figure 4.**
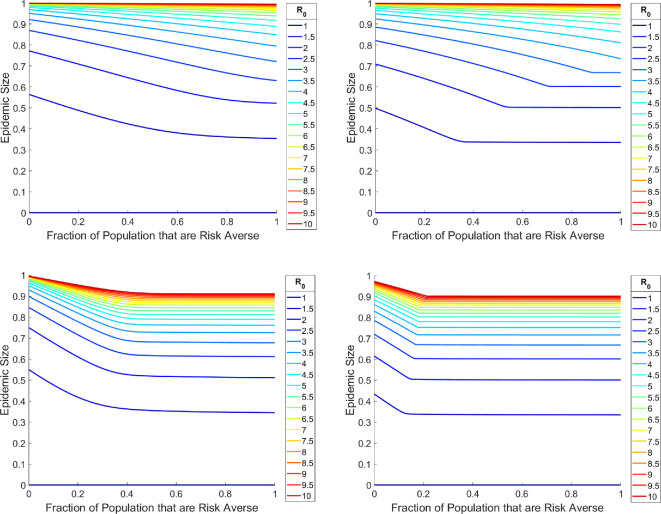
Final epidemic size versus fraction of population that are risk-averse ($S_{1}$). Simulations in the left column have a higher δ than simulations in the right column. Parameter values: $R_{0}=\beta ,\gamma =1,\lambda_{1}=100,\lambda_{2}=1$. Initial conditions: $I(0)=10^{-8},P_{1}(0)=P_{2}(0)=R(0)=0$.

The transition from the overdamped regime to the underdamped regime is sharper (graphically, the transition is where the elbow bend occurs in figure 4) when the usage of interventions is prolonged (or equivalently when the rate at which protected individuals relax back into the regular susceptibility classes decreases). Consider the following scenario which is identical to the previous setup, except now the intervention reversion rate ($\delta P_{i}$) has been reduced by an order of magnitude (figure 4). This corresponds to a scenario where people continue to use the intervention (i.e. such as wearing masks or social distancing) on a timescale significantly longer than the transmission timescale ($\frac{1}{\delta }>>\frac{1}{\beta }$).

This suggests that increasing the timescale at which individuals continue to use interventions decreases the fraction of risk-averse individuals needed to achieve the same epidemic size. This is reflected in the horizontal shift of the transition region to the left when comparing figures in the left column and the right column (figure 4). We also briefly investigate what happens if we allow for heterogeneous relaxation rates between the difference groups, which is more reflective of what occurs in reality, finding the epidemic size to be more attune to the slower relaxation time scale (electronic supplementary material).

#### 2.1.4. Increasing heterogeneity in risk tolerance can either increase or decrease the epidemic size

There are many types of heterogeneity that may be present in the population, arising from differences in characteristics such as biological, cultural factors and socioeconomic factors. The effect of each type of heterogeneity on disease dynamics is an area of active study. Increasing heterogeneity in the population through increasing the variation in their contact patterns, age or general susceptibility can impact features of the epidemic dynamics such as the herd immunity threshold [48–51].

**Figure 5.**
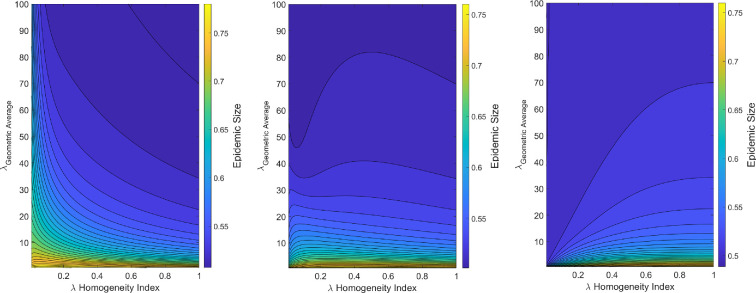
Epidemic size under differing levels of heterogeneity in the adoption rate for interventions. The mean adoption rate of the two groups (i.e. geometric average of $\lambda_{1},\lambda_{2}$) is compared to the difference in the two adoption rates as parameterized by a homogeneity index (see §4 for definition). *Left* is when the fraction of the population with low-risk tolerance ($x_{1}$) is 0.2; *centre* is when $x_{1}=0.35$; and *right* is when $x_{1}=0.5$. Parameter values: $\beta =2,\gamma =1,\epsilon =0.7,\delta_{1}=\delta_{2}=0.5$. Initial conditions: $I(0)=10^{-8},P_{1}(0)=P_{2}(0)=R(0)=0$.

We find in this model of heterogeneous behaviour that it is possible to switch from a regime where increasing the heterogeneity in risk tolerance results in a decrease in epidemic size to a regime where increasing the heterogeneity in risk tolerance results in an increase in epidemic size. This result also does not have to necessarily be due to a dramatic shift in parameters. From figure 5, we see this shift can arise from solely varying the fraction of the population with low-risk tolerance by a small amount.

The intuition underlying this result is that when the average adoption rate ($\lambda_{average}$), which is expressed as a (geometric or arithmetic) weighted mean of the adoption rates of the two groups, is fixed at a constant level, then the epidemic size can be suppressed through either varying the fraction of the population in each group or through varying each group’s adoption rate. When risk-averse people make up a smaller fraction of the population than risk-taking people, then it would be more beneficial in reducing epidemic size to have the adoption rates of the two groups be more similar (i.e. more homogeneous) as that would imply risk-taking people (which are then the majority of the population) would have a similar adoption rate to risk-averse people. As an increasingly larger fraction of the population is composed of risk-averse people, then it becomes increasingly beneficial in reducing epidemic size to have the adoption rates of the two groups be more different (i.e. more heterogeneous) as the deleterious effects of highly risk-taking people (which are then the minority of the population) can be mitigated by the large presence of risk-averse people.

#### 2.1.5. Epidemic size does not necessarily decrease with an increasing fraction of risk-averse people

General intuition would suggest that as one increases the fraction of risk-averse people in the population that the overall epidemic size would go down. However, the introduction of the adaptive behaviour mechanism allows for regimes where this is no longer strictly the case. Thus, it is no longer a guarantee that decreasing the population’s overall risk tolerance will always improve epidemic outcomes.

**Figure 6.**
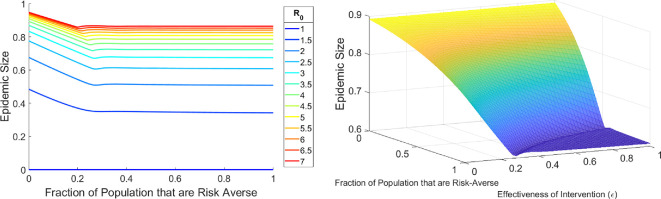
(a) Final epidemic size as a function of the proportion of the population that are risk-averse ($S_{1}$). Parameter values: $\lambda_{1}=10,\lambda_{2}=0.5,\epsilon =1,\delta_{1}=\delta_{2}=0.1$. Initial conditions: $I(0)=10^{-7},P_{1}(0)=P_{2}(0)=0,R(0)=0$. (b) Same as in (a) except now the effectiveness of the intervention (ϵ) is allowed to vary.

Consider figure 6a, where we find a small region after the transition to the underdamped regime where there is an increase in the epidemic size when increasing the fraction of the population that are risk-averse.

If we also vary the effectiveness of the intervention as an additional axis (figure 6b), we observe that there is a small trench in the threshold region surrounding the plateau area. This double descent suggests that the landscape can potentially be quite complicated when risk tolerance in the population is partitioned into even more groups.

## 3. Discussion and conclusions

In this article, we have proposed a simple model to model heterogeneity in risk tolerance levels in the population. We find that including a behavioural mechanism for adopting interventions that adapts with the level of infections greatly expands the variety in epidemic dynamics and outcomes that can occur.

The general picture from the findings suggests that epidemic dynamics under adaptive intervention adoption fall into either an underdamped regime or an overdamped regime. The underdamped regime has a special property in which the epidemic size equals the herd immunity threshold exactly, which means no epidemic overshoot occurs. The system can be driven into this regime when protection mechanisms (such as numbers of risk-averse people, intervention effectiveness and duration of intervention usage) are increased to a sufficiently high level. This regime is also marked by damped oscillations in the phase space of infecteds and susceptibles. In direct contrast, the overdamped regime closely resembles the dynamics of a simple SIR model without behaviour, in which there are no oscillations and a non-zero overshoot [52], which makes the epidemic size greater than the herd immunity threshold. While we have limited our numerical exploration of the model to two groups of different risk-tolerance levels, in principle our framework can accommodate an arbitrary number of risk-tolerance groups. While the combinatorial increase in parameter space makes it difficult to thoroughly explore larger models, we might naively expect there to be a complex landscape of outcomes, which might reveal other counter-intuitive phenomenology akin to the non-monotonic dependence of epidemic size with increasing fraction of risk-averse people for two groups.

We have looked for some evidence in the historical data on outbreaks for these damped oscillations due to cycles in adoption and relaxation of interventions. While such data are in very limited supply, previous analysis suggested that relaxation of social distancing measures may have led to multiple waves of infection in the Spanish flu of the early twentieth century [53,54]. Dating back to the time of the bubonic plague, there are data from an outbreak in 1636 in the parish of St Martin in the Fields, which showed how relaxation of quarantining and isolation measures lead to a smaller secondary wave of infections [55]. More recently, premature relaxation of interventions during the COVID pandemic might have led to secondary outbreaks [56]. The complex milieu of disease, biological, behavioural, political, economic and cultural factors however make it difficult to isolate cause-and-effect in real-world epidemics [57].

The results here are reminiscent of feedback control systems commonly studied in control theory. Here, the set point is the herd immunity threshold, which is determined by the basic reproduction number ($R_{0}$). The ability of the population to reach this set point for epidemic size without additional overshoot depends on the effectiveness of the feedback mechanism from coupling intervention usage to the fraction of infected people. In the model presented, the adoption of interventions is a continuous process, in which the different groups are constantly reacting to the level of infections without requiring any notion of time or thresholds. In contrast, existing research on mitigation has considered more active control where activation and intervention timing play a key role [58–60]. Future work may explore how to synergistically utilize both active and continuous mechanisms for control.

The inclusion of heterogeneity in risk tolerance and adaptive adoption of interventions leads to several unexpected conclusions. We find that increasing heterogeneity in risk tolerance levels in the population can lead to either an increase or decrease in the epidemic size. The direction of the trend depends nonlinearly on the composition of the population in terms of the ratio of risk-averse to risk-taking individuals and their respective intervention adoption rates. This adds to a small literature that demonstrates how heterogeneity can actually lead to a larger epidemic [36,61].

Interestingly, these results on heterogeneity also can be used to address the question of whether distributed or centralized control of mitigation results in a smaller outbreak. Control of factors such as mobility may be more practically achieved in a more centralized and unified fashion [62,63], whereas a distributed approach may be more appropriate when considering factors such as speed and individual agency. In a centralized scenario, a single entity controls the dynamics. That situation has an exact correspondence to the homogeneous population considered here, where all individuals respond in unison. Distributed control allows for more localized control, such as individuals or small groups deciding if they want to mask or social distance. This corresponds to the heterogeneous scenarios considered here, where there are multiple groups each with differing risk tolerance level. The results suggest that in some scenarios a single, coordinated response would be better for mitigation, whereas in other parameter regimes, a more decentralized strategy would be more optimal.

We also find that increasing overall protection mechanisms does not always result in a monotonic decrease in epidemic size. In scenarios when the adoption rate begins to approach the transmission rate, near the critically damped boundary a nonmonotonicity can arise. This suggests that when intervention usage and effectiveness are tenuous, the dynamics become more complex and predicting what epidemic outcomes will result becomes significantly more difficult. Understanding how these nonlinear effects combine with other biological and behavioural heterogeneities will be important to explore in future work. Despite showcasing a variety of potential behaviours, the model considered here is fundamentally a deterministic one. It will be interesting to explore what additional regimes of outcomes occur when stochastic effects are considered. Additional complexities can also be considered in future work, such as the impact of spatial heterogeneity and behavioural changes in individuals that have become infected.

## 4. Methods

### 4.1. Defining the λ homogeneity index

The λ homogeneity index is defined as follows. We will assume the initial condition that at the beginning of the dynamics, the total population is composed of the fraction of the population in the low-risk tolerance group $x_{1}$, the high-risk tolerance group $x_{2}$ or the infected class. We will assume the fraction of the population initially infected is sufficiently small so that the size of the two susceptible compartments is given by $x_{1}$ and $1-x_{1}$, respectively.

Define a homogeneity index parameter (c) that captures the degree of homogeneity between the two adoption rates, $\lambda_{1}$ and $\lambda_{2}$. We define the parameter domain to be the unit interval, c∈(0,1]. An index value of c=1 indicates $\lambda_{1}=\lambda_{2}$, while decreasing the index value towards 0 increases the difference between $\lambda_{1}$ and $\lambda_{2}$.

We can define the average adoption rate as being either a geometric or arithmetic mean of the two adoption rates. The choice one makes is arbitrary, so we present prescriptions for both routes. In both cases, we will map the level of homogeneity to the unit interval.

#### 4.1.1. Geometric average

Let the geometric average of the two adoption rates be given by $\lambda_{\text{Geometric Average}}$:


(4.1) $$\lambda_{\text{Geometric Average}}\equiv \lambda_{1}^{x_{1}}\lambda_{2}^{1-x_{1}}.$$

We assume the following scalar relationship between $\lambda_{2}$ and the average λ:


(4.2) $$\lambda_{2}=c\lambda_{\text{Geometric Average}},c\in (0,1].$$

These two equations combine to give the following equation for $\lambda_{1}$:


(4.3) $$\lambda_{1}=\frac{\lambda_{\text{Geometric Average}}}{c^{\frac{x_{1}}{1-x_{1}}}},c\in (0,1].$$

#### 4.1.2. Arithmetic average

Let the arithmetic average of the two adoption rates be given by $\lambda_{\text{Arithmetic Average}}$:


(4.4) $$\lambda_{\text{Arithmetic Average}}\equiv x_{1}\lambda_{1}+(1-x_{1})\lambda_{2}.$$

We assume the following scalar relationship between $\lambda_{2}$ and the average λ:


(4.5) $$\lambda_{2}=c\lambda_{\text{Arithmetic Average}},c\in (0,1].$$

These two equations combine to give the following equation for $\lambda_{1}$:


(4.6) $$\lambda_{1}=\frac{\lambda_{\text{Arithmetic Average}}(1-x_{1}c)}{1-x_{1}},c\in (0,1].$$

Again, an index value of 1 indicates $\lambda_{1}=\lambda_{2}$, while decreasing the index value towards 0 increases the difference between $\lambda_{1}$ and $\lambda_{2}$.

#### 4.1.3. Homogeneity index in larger groups

The above parameterization of homogeneity is useful for the setting of a two-group model because the entirety of the possible difference in heterogeneity between $\lambda_{1}$ and $\lambda_{2}$ can be mapped to the unit interval. This allows us to more easily see the qualitative shift in trend in phenomenology that prompted this section of text in the first place. An alternative parametrization might have used the variance between the two adoption rates. However, the domain of such a parameterization is unbounded, making it impossible to explore fully numerically. Moving beyond two groups would necessitate a different parameterization (such as through using a variance or coefficient of variation approach).

## Data Availability

The code used to generate all figures is provided in the electronic supplementary material [64].
